# Screening and Selection of a New Medium for Diosgenin Production via Microbial Biocatalysis of *Fusarium* sp.

**DOI:** 10.3390/ph14050390

**Published:** 2021-04-21

**Authors:** Wancang Liu, Haibo Xiang, Tao Zhang, Xu Pang, Jing Su, Hongyu Liu, Baiping Ma, Liyan Yu

**Affiliations:** 1 Division for Medicinal Microorganisms Related Strains, CAMS Collection Center of Pathogenic Microorganisms, Institute of Medicinal Biotechnology, Chinese Academy of Medical Sciences & Peking Union Medical College, Beijing 100050, China; liuwancang@imb.pumc.edu.cn (W.L.); zhangt@cpcc.ac.cn (T.Z.); pangxu@imb.pumc.edu.cn (X.P.); sujing@cpcc.ac.cn (J.S.); hyliu@cpcc.ac.cn (H.L.); 2 State Key Laboratory of Biocatalysis and Enzyme Engineering, School of Life Sciences, Hubei University, Wuhan 430011, China; xhb2086@hubu.edu.cn; 3 Beijing Institute of Radiation Medicine, Beijing 100850, China; mabaiping@sina.com

**Keywords:** diosgenin, microbial biocatalysis, medium compositions, *Dioscorea zingiberensis* C. H. Wright, endophytic fungus, *Fusarium* strain, Plackett–Burman design, Box–Behnken design, submerged fermentation

## Abstract

Steroidal saponins are widely used as starting precursors and medical intermediates for the semi-/total-synthesis of hundreds of steroidal drugs. One such steroidal saponin is diosgenin, which has attracted significant attention due to the huge market demand in the pharmaceutical industry. Due to water waste and severe environmental pollution, the traditional diosgenin production process based on direct acid hydrolysis is no longer used. In this study, to develop a submerged fermentation (SmF) medium for clean diosgenin production via efficient microbial biocatalysis, the Box–Behnken design (BBD) in combination with the Plackett–Burman design (PBD) was used to determine the medium compositions for *Fusarium* strains. Three components (wheat bran, phosphate, and Tween-80) were determined as significant factors by the PBD. Using the BBD, the three significant factors were further optimized, and the optimum values were determined for maximal diosgenin production. With 21.16 g/L of wheat bran, 9.60 g/L of phosphate, and 1.97 g/L of Tween-80, the diosgenin yield was 2.28%, i.e., 3.17 mg/L/h. The experimental values agreed with the predicted values, representing a significant increase in diosgenin production compared to its production using the basic SmF medium. For the first time, we reported the development of a new medium for *Fusarium* strains to produce diosgenin via microbial biocatalysis of the root of *Dioscorea zingiberensis* C. H. Wright (DZW). A simple-composition, low-cost, and high-efficiency medium was developed for the first time for the SmF of *Fusarium* strains. The medium is considered useful for large-scale SmF and may be applicable to other fungi. This study lays a solid foundation for diosgenin production in an acid-free and wastewater-free way. It may also provide fundamental support for producing other value-added products via microbial biocatalysis of low-value materials by endophytic fungi.

## 1. Introduction

**Figure 1:**
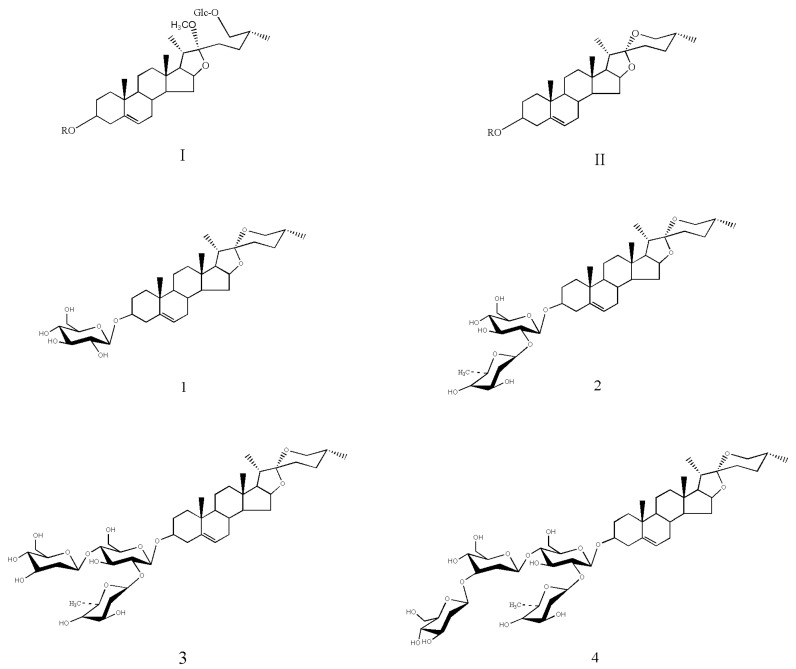
Chemical structures of steroidal saponins in plants. The steroidal saponins were mainly divided into two types, furostanol-type (**I**) and spirostanol-type (**II**). Spirostanol-type saponins in DZW primarily include trillin (**1**), prosapogenin A of dioscin (**2**), deltonin (**3**), and zingiberensis newsaponin (**4**).

*Dioscorea zingiberensis* C. H. Wright is extensively used as a well-known traditional Chinese medicine (TCM). The plant has also been used as food material by the Chinese for thousands of years [1]. Recently, more than 100 steroidal saponins were purified from *D. zingiberensis* and characterized. According to the structural diversity at *O*-26 and *C*-22 positions, steroidal saponins are divided into two types (Figure 1), including spirostanol–type and furostanol type [2,3]. After dehydration–condensation between *C*-26 and *C*-22, the sixth ring is formed in spirostanol-type saponins. Furostanol-type saponins are determined by a semiacetal at the *C*-22 position to afford a five-ring system [4]. Furostanol-type saponins can be converted to spirostanol-type saponins under an uncontrolled process [5]. Conversely, glycosyltransferase can convert spirostanol-type saponins to their furostanol types under specific reaction conditions [4]. Pharmacodynamic studies on *D. zingiberensis* have been widely explored, especially in terms of the biological activities of various steroidal saponins isolated from this TCM. A variety of beneficial bioactivities have been ascribed to steroidal saponins, including raising coronary flow, acting as anti-inflammation agents, protecting cardiac muscle, lowering blood pressure, protecting against anti-myocardial ischemia, and inhibiting platelet aggregation [6,7,8,9].

Deglycosylation at the *C*-3 position of spirostanol-type steroidal saponins yields diosgenin (25R-Spriost-5-en-3β-OH), which is an essential steroidal saponin in the pharmaceutical industry. Diosgenin is the most preferred starting material for the commercial synthesis of oral contraceptives, sex hormones, and other steroidal drugs [10]. Due to the high content of steroidal saponins (2 to 3%, *w*/*w*; chemical structures are shown in Figure 1) in the *D. zingiberensis* C. H. Wright tuber (DZW) [11], DZW is the primary raw resource for industrial diosgenin production. Over 1000 tons of diosgenin are produced every year, and the high demand for diosgenin increases year on year [12,13]. Traditionally, diosgenin is prepared by direct acid hydrolysis of DZW [14]. Surprisingly, saponins in plant cells are wrapped tightly in large amounts of starch and fiber. High concentrations of starch (30 to 40%, *w*/*w*) and fiber (40 to 50%, *w*/*w*) in plants leads to large amounts of wastewater and serious environmental pollution when the conventional diosgenin production process is implemented [15,16]. In contrast, starch and fiber block the effective access of the catalyst for efficient transformation of saponins into diosgenin. Considering the particular form in which diosgenin exists in plants and the apparent issues of acid hydrolysis, progress has been made to develop clean methods for efficient diosgenin production [17,18,19,20,21], such as removing starch and fiber before acid hydrolysis, recovering starch and fiber after acid hydrolysis, and disposing of sewage after acid hydrolysis. However, the acid is still required, and there is a long way ahead to meet the needs for a low-cost, pollution-free, and energy-saving production process. In recent years, the transformation base of microbial biocatalysis has become increasingly attractive because the acid is replaced by enzymes to cleave the glycosidic bond of saponins in DZW [22,23,24]. Microbial biocatalysis is likely a promising alternative to diosgenin production in industrial applications.

In our previous work, hundreds of endophytic fungi were purified from various plants (www.cpcc.ac.cn (accessed on 24 March 2021)). The strains that belonged to the *Fusarium* genus could biocatalyze DZW and produce diosgenin. A new solid-state fermentation (SSF) process was then developed for clean diosgenin production [25]. Due to the advantages of submerged fermentation (SmF) technology [26], further processes are also being developed [25,27]. Herein, the fermentation medium is one of the most important factors because the medium composition can significantly affect the strain’s growth and production levels. Medium optimization is also required for future industrial applications of the bioprocess. After optimization of culture medium components, an ~three-fold improvement in the extracellular expression of cellulases such as endoglucanase II in BL21 (DE3) pLysS was observed [28]. The exopolysaccharides yield from *Lactobacillus rhamnosus* increased from 85 to 1138 mg/L when an optimized medium was used [29]. Medium optimization led to an ~36% increase in acetoin production [30], and significantly improved the production of antibacterial peptide in *Bacillus licheniformis* [31]. Optimal media can enhance the production of both macromolecules and small molecules from natural materials as well as artificial products. To the author’s best acknowledge, media for *Fusarium* spp. to produce diosgenin via SmF are extremely limited. Therefore, it is necessary to develop an optimal medium for clean diosgenin production via efficient microbial biocatalysis using *Fusarium* spp. in SmF processes.

The objective of this work was to develop a medium for clean diosgenin production via the efficient SmF of *Fusarium* to decrease the production cost, increase production efficiency, and fully realize the industrial preparation of diosgenin. By taking *Fusarium* sp. CPCC 400226 as a typical example, the Box–Behnken design (BBD), in combination with the Plackett–Burman design (PBD), was used to determine the medium compositions, and a new SmF medium was developed via the microbial biocatalysis of *Fusarium* strain. Not only can the fermentation medium be used for diosgenin production from DZW, it may be also fundamental and useful for other endophytic fungi to generate value-added products. This study lays a solid foundation for diosgenin production in a sustainable way and therefore provides practical support for industrial applications of microbial biocatalysis by endophytic fungi.

## 2. Results

### 2.1. Selection of Primary Medium Components

The PBD method was used to screen and select the most significant independent variables that notably affect the diosgenin yield. Seven factors (Tween-80, vitamin, amino acid, PTM4, yeast extract, wheat bran, and phosphate) were tested in 12 runs of the PBD. As shown in the matrix (Table 1), experiments based on the PBD showed a wide variation in the diosgenin yield, from 0.11% to 1.99%. The selected medium components significantly affected diosgenin production. The highest response was observed in run 1, followed by runs 8, 4, and 9. At a high concentration of wheat bran in the medium, the diosgenin yield was high. In contrast, the lowest response was observed in run 7, followed by runs 12 and 11. At a low concentration of phosphate in the medium, the low diosgenin yield was low.

Table 2 represents the analysis of variance (ANOVA) results of the PBD model (*p* < 0.05). Wheat bran was determined as the most significant factor, with an *F*-value, *p*-value, and contribution ratio of 210.1, 0.0001, and 62.9%, respectively. The other significant factors were phosphate and Tween-80. These three variables positively affected the diosgenin yield, with an SS of 3.03, 1.40, and 0.28, respectively. Vitamin, amino acid, PTM4, and yeast extract were insignificant factors in the PBD. In addition, the PBD model was significant with an *F*-value of 47.1 and a *p*-value of 0.0011. The R^2^ (coefficient of correlation), predicted R^2^, and adjusted R^2^ reached 0.9880, 0.9670, and 0.8921, respectively.

**Figure 2:**
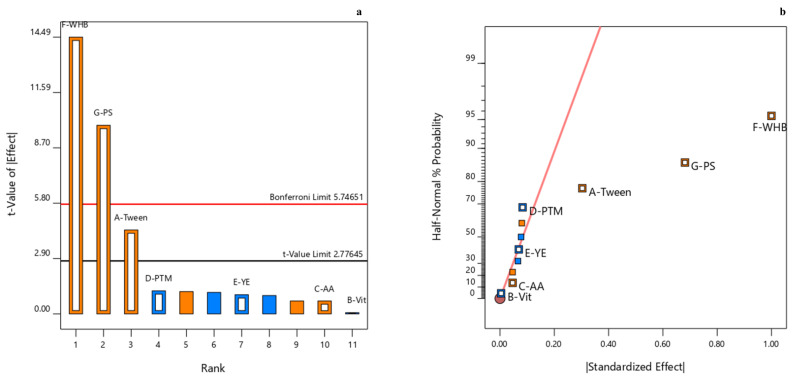
The PBD plots: Pareto chart (**a**) and half-normal probability plot (**b**). The positive effects and negative effects are indicated by yellow points and blue points, respectively. Factors *A*, *B*, *C*, *D*, *E*, *F*, and *G* represent Tween-80, vitamin, amino acid, PTM4, yeast extract, wheat bran, and phosphate, respectively.

A Pareto chart and a half-normal probability plot were created to identify significant factors affecting the microbial biocatalysis of DZW via SmF of *Fusarium* sp. CPCC 400226. As shown in Figure 2a, the Bonferroni limit line with a value of 5.75 and the *t*-value limit line with a value of 2.78 are obtained. The *t*-value of wheat bran and phosphate was above the Bonferroni limit line, while that of wheat bran, phosphate, and Tween-80 was above the *t*-value limit line. In the half-normal probability plot (Figure 2b), five of the seven examined variables (i.e., wheat bran, phosphate, Tween-80, amino acid, and vitamin) had a positive effect on the diosgenin yield. The most significant variable was wheat bran, followed by phosphate and Tween-80. Wheat bran had the highest enhancement effect on the diosgenin yield from DZW via the SmF of *Fusarium* sp. CPCC 400226. The highest negative factor was PTM4; however, it had no significant impact on the diosgenin yield. Based on the above investigation, the primary medium components were determined as wheat bran, phosphate, and Tween-80.

### 2.2. Optimization of Medium Compositions

#### 2.2.1. Model Building

The BBD method was used to build the model and identify mutual factor interactions. Three factors (wheat bran, phosphate, and Tween-80) were tested in 15 runs of the BBD. As shown in the matrix (Table 3), experiments based on the BBD showed a wide variation in the diosgenin yield, from 0.37% to 1.98%. The highest response was observed in run 13, followed by runs 12, 2, and 8. In contrast, the lowest response was observed in run 15, followed by runs 7, 10, and 5.

#### 2.2.2. Mathematical Validation

Based on the ANOVA results of the BBD model (*p* < 0.05), the following second-order polynomial equation was obtained:

*Y* (%, diosgenin yield) = 1.96 + 0.52*X*_1_ + 0.26*X*_2_ + 0.034*X*_3_ − 0.15*X*_1_*X*_2_ − 0.14*X*_1_*X*_3_ − 0.0023*X*_2_*X*_3_ − 0.64*X*_1_^2^ + 0.0054*X*_2_^2^ − 0.25*X*_3_^2^ (1)

where *Y* is the diosgenin yield (%) and the intercept was 1.96. The linear coefficients were 0.52, 0.26, and 0.034; the interactive coefficients were −0.15, −0.14, and 0.0023; and the quadratic coefficients were −0.64, 0.0054, and −0.25.

As shown in Table 4, the three selected variables were significant, with *F*-values of 3431.2, 883.0, and 14.5, respectively, and *t*-values of <0.0001, <0.0001, and 0.0121, respectively. The mutual interactions between wheat bran and phosphate and between wheat bran and Tween-80 had a significant effect on the microbial biocatalysis of DZW via the SmF of *Fusarium* sp. CPCC 400226. The lack of fit was not significant with an *F*-value of 1.0 and a *p*-value of 0.5465. In addition, the BBD model was significant, with an *F*-value of 812.8 and a *p*-value of <0.0001. The R^2^, predicted R^2^, and adjusted R^2^ reached 0.9993, 0.9981, and 0.9929, respectively.

#### 2.2.3. Mutual Interactions Analysis

**Figure 3:**
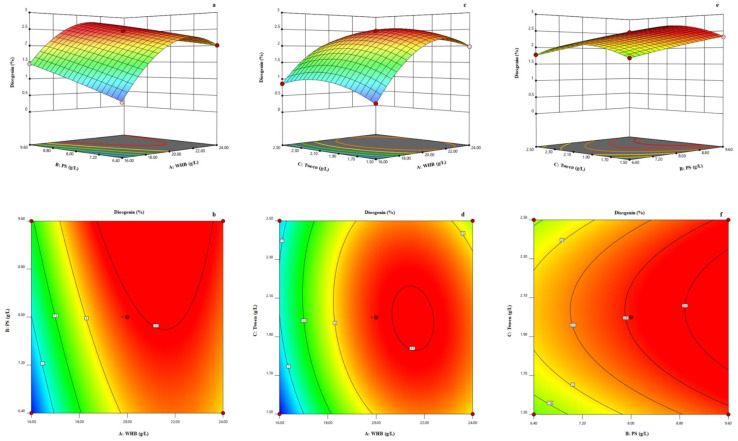
Contour maps and response surface plots in the BBD. (**a**) Mutual interaction between wheat bran and phosphate based on response surface plot. (**b**) Mutual interaction between wheat bran and phosphate based on contour map. (**c**) Mutual interaction between wheat bran and Tween-80 based on response surface plot. (**d**) Mutual interaction between wheat bran and Tween-80 based on contour map. (**e**) Mutual interaction between phosphate and Tween-80 based on response surface plot. (**f**) Mutual interaction between phosphate and Tween-80 based on contour map.

The contour maps and response surface plots were plotted for mutual factor interactions analysis. Figure 3a,b shows the effect of wheat bran (*X*_1_) and phosphate (*X*_2_) on the diosgenin yield, with Tween-80 (*X*_3_) being maintained at a constant zero level (level zero, 2 g/L). As the wheat bran concentration increased, the diosgenin yield first sharply increased and then gradually decreased. The mutual interaction between wheat bran (*X*_1_) and Tween-80 (*X*_3_) is shown in Figure 3c,d, with phosphate (*X*_3_) being maintained at a constant zero level (level zero, 8 g/L). Figure 3e,f shows the effect of phosphate (*X*_2_) and Tween-80 (*X*_3_) on the diosgenin yield, with wheat bran (*X*_1_) being maintained at a constant zero level (level zero, 20 g/L). As the concentration of phosphate in the medium increased, the diosgenin yield gradually increased to the maximum; however, with an increasing concentration of Tween-80, the diosgenin yield first increased and then gradually decreased.

#### 2.2.4. Optimum Condition Selection

Using the BBD in combination with the PBD (PBD–BBD), the main medium components were determined as 20.9 g/L of wheat bran, 8.4 g/L of phosphate, and 1.9 g/L of Tween-80. By taking *Fusarium* sp. CPCC 400226 as an example, a new medium for clean diosgenin production through efficient microbial biocatalysis of *Fusarium* spp. was developed. The final medium compositions were as follows: 1.9 g of Tween-80, 1.0 g of vitamin, 1.0 g of amino acid, 1.25 g of PTM4, 10 g of yeast extract, 20.9 g of wheat bran, and 8.4 g of phosphate per liter. Through microbial biocatalysis of DZW by *Fusarium* sp. CPCC 400226 with this optimized medium, the predicted diosgenin yield was 2.28%.

### 2.3. Experimental Validation

**Figure 4:**
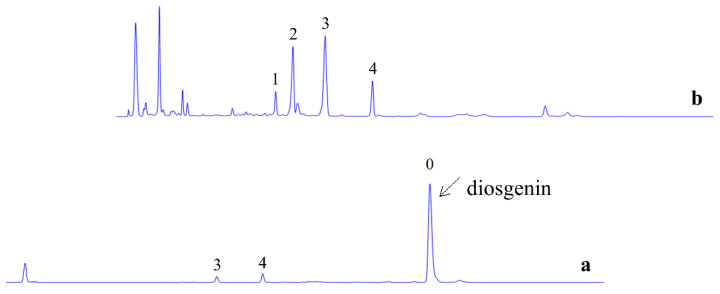
HPLC-ELSD analysis of products and substrates. (**a**) Profile of diosgenin from DZW. The arrow indicates the product of diosgenin (0). The main residuals were prosapogenin A (3) and trillin (4). (**b**) Profile of DZW before microbial biocatalysis. DZW was mainly composed of zingiberensis newsaponin (1), deltonin (2), prosapogenin A (3), and trillin (4).

The SmF of *Fusarium* sp. CPCC 400226 was conducted using the new medium obtained from the PBD–BBD. As seen in Figure 4, the DZW were efficiently transformed via microbial biocatalysis. Zingiberensis newsaponin and deltonin were almost transformed, and their conversion rate was more than 95%. In the final post-reaction broth, slight amounts of prosapogenin A and trillin were observed. The experimental diosgenin yield was 2.23 ± 0.16%, which was equal to 3.10 ± 0.23 mg/L/h.

## 3. Discussion

SmF technology has been extensively used to produce various pharmaceutical chemicals, such as antibiotics, polyunsaturated fatty acids, and vitamins [32,33,34]. Recently, many vital enzymes and natural products were obtained from the fermentation broth of fungi, although fungal fermentation is still a complex bioprocess with numerous concerns [35]. However, the original production cannot always sustainably meet industrial purposes. Strategies based on the genetic makeup of the strains, the manipulating of nutritional requirements, and environmental conditions are widely used for enhanced production of these products. Developing a fermentation medium is an essential step in improving the bioprocess performance without increasing cost; it determines growth and productivity. Most fungi have a strong requirement for oxygen to maintain growth and synthesize metabolites, but media containing large quantities of organic and inorganic components may cause a lack of dissolved oxygen. More importantly, medium components are important for the expression of secreted proteins, which have a significant effect on the performance of microbial biocatalysis in SmF. To the best of our knowledge, there is little information about the role of factors and their levels in controlling the SmF of *Fusarium* strains, especially for diosgenin production. In contrast, medium development is still one of the most critical tasks that are performed before any large-scale production and involves many challenges. In this case, it is necessary to develop a simple-composition, low-cost, and high-efficiency medium for *Fusarium* strains to produce diosgenin in SmF.

First, the effects of various nutritional requirements on the diosgenin yield from the microbial biocatalysis of DZW by the tested *Fusarium* strain were examined. In the traditional medium development technique, the methodology based on one-factor-at-a-time (OFAT) experiments is still in use, which mainly includes experiments of supplementation, removal, and replacement. Considering the deficiencies and shortcomings of OFAT [36], the statistical medium development technique of PBD was used to determine the primary medium components. The PBD is a good “screening design” to determine the relative significance of each medium component for a specific output. Prior to PBD experiments, pre-investigation on the microbial biocatalysis of DZW by *Fusarium* sp. was conducted. DZW was suspended in water, and the resulting mixture was subjected to SmF. However, the yield of the newly produced diosgenin was close to zero, although we assumed that the nutrients in DZW could successfully support the growth of the strain and, therefore, produce diosgenin. Based on literature research [37,38,39,40] and the investigation conducted in this laboratory [25], various medium components were followed, such as supporting material (wheat bran vs. rice husk), nitrogen source (yeast extract vs. peptone), surfactant (Tween-80 vs. Triton X-100), and trace elements (PTM vs. tropical marine). Of these, wheat bran, yeast extract, Tween-80, and PTM showed better performance with regard to diosgenin production (data not shown). Therefore, these four factors and the other three factors (vitamin, amino acid, and basic salts) were subjected to the PBD. We found that wheat bran, phosphate, and Tween-80 were the main medium components for diosgenin production via the microbial biocatalysis of *Fusarium* strains by SmF.

Due to the favorable consistency and suitable particle size, wheat bran is a better support material and is widely used in SSF [40]. Keeping this in mind, wheat bran was selected for the PBD experiment. As surmised, wheat bran provided good porosity for microorganism growth and substrate consumption, which made proper enzyme excretion possible and, therefore, realized efficient microbial biocatalysis of DZW by *Fusarium* sp. CPCC 400226. In addition, wheat bran can be easily obtained in many residual natural materials obtained from agricultural activities. It has a low cost and was the chosen carbon source for diosgenin production. Phosphate is the required component for forming nucleic acids and phospholipids in microbial cells. Conversely, excessive phosphate highly influences and even inhibits the production of secondary metabolites [41,42]. We found that phosphate was a required component in the SmF medium for diosgenin production via the microbial biocatalysis of DZW. In this case, phosphate might be the fundamental nutrient for better growth of *Fusarium* strains. Phosphate probably influenced and even promoted the biosynthesis of glycosidase, which could complete the deglycosylation of the steroidal saponins in DZW. To improve the production of desired products, surfactants were added to the fermentation medium in SmF [43,44,45]. We assumed that a specific amount of surfactant might improve the cell membrane permeability by affecting the permeation barrier. The surfactant Tween-80, structurally similar to phosphatide molecules in the cell membrane, was another main factor that significantly affected the SmF of *Fusarium* strains to produce diosgenin via the microbial biocatalysis of DZW. The production of enzymes and chemicals in SmF broth is highly regulated by the surfactant, although it is not clear how Tween-80 influences diosgenin formation in the SmF process of *Fusarium* sp. CPCC 400226. One assumption is that Tween-80 either facilitates the invasion of saponins into the cells or stimulates the release of enzymes from the aerobic fungus.

Second, the BBD was used to identify the optimum level of each significant medium component. Based on the PBD experiments, the SmF bioprocess was optimized. In contrast to the conventional method, the statistical method has many advantages [46,47]. Particularly, the significant effect of each factor and their mutual interactions can be effectively determined by the statistical method. To assess the adequacy of the model, various diagnostic plots were created (Figure S1). It is suggested that the experimental values are in good agreement with the predicted values, because data points in the alignment plot of these two groups were reasonably aligned (Figure S1a). Meanwhile, normal distribution was observed in the normal percentage probability plot (Figure S1b), which verified the accuracy of the model. It is further implied that the model is robust. The absolute values of internally studentized residuals indicates that the model is adequate, which was clarified by the low values (<3) of each data point in the plot of internally Studentized residuals (Figure S1c). Moreover, the perturbation plot was depicted in the BBD (Figure S2). The results obtained from these plots were in good agreement with each other. Following the contour maps and response surface plots clarified that the BBD model not only possesses satisfactory fits for the diosgenin yield but also reliably fits the interactions between various variables.

Finally, the reliability of the experimental model using a combined PBD–BBD was validated. Under optimum conditions in SmF medium, the experimental diosgenin yield was in good agreement with the predicted value from the model (2.23% vs. 2.28%). Due to the differences in the sources of DZW, the natural contents of total steroidal saponins in DZW often vary. In the present study, an ~85% DZW conversion rate was obtained when 40 g/L of DZW was fermented using the newly developed medium. Meanwhile, slight residual prosapogenin A of dioscin and trillin were detected in the final fermentation broth of *Fusarium* sp. CPCC 400226, which might be due to the steric effect of substituent groups at the *C*-3 position, because these groups are much more challenging to hydrolyze [48]. In addition, the validity of the statistical design was confirmed by the good data agreement in the PBD–BBD model, and along with high reliability, precision, and adequacy. Overall, the model is workable. More importantly, a simple-composition, low-cost, and high-efficiency medium has been established for the first time for the SmF of *Fusarium* strains to produce diosgenin. Based on screening and selection of the most important components in the SmF medium, the combined statistical tool of the PBD–BBD was successfully used to simplify the medium components and improve the bioprocess development efficiency. Ultimately, a new medium was developed for the SmF of *Fusarium* strains that significantly improved the diosgenin yield via the microbial biocatalysis of DZW.

Based on the above-mentioned investigation, a new SmF medium was developed for the first time for *Fusarium* strains. As a continuation of the present study, the medium is being used for larger-scale fermentation with bioreactors. The strain grew well, and diosgenin was produced in 5-L bioreactors, laying down a solid foundation for the pilot-scale production of diosgenin. Moreover, the successful bioreactor-scale SmF further verified the reliability of the statistical model and the biocatalytic efficiency of the newly developed medium. In addition to medium components, some environmental conditions can also significantly affect fungal growth and production [36]. Of these, the dissolved oxygen level and aeration in bioreactors are often specifically controlled and monitored, because their effects on the production efficiency cannot be fully understood in the common shake flasks. In addition, the results suggest that the addition of antifoam reagent, culture time, and temperature significantly affect the performance of the bioprocess when this newly developed medium was used to produce diosgenin by the SmF of *Fusarium* strains. As expected, this medium has promising application prospects and can help draw a blueprint for clean diosgenin production via efficient microbial biocatalysis.

## 4. Materials and Methods

### 4.1. Materials and Strain

Dried DZW and wheat bran were from Ankang (Shanxi, China) and Huining (Gansu, China), respectively. Tween-80 and amino acid were purchased from Yuanye Biotechnology Co., Ltd. (Shanghai, China). Vitamin was purchased from Wyeth Pharmaceutical (China) Co., Ltd. (Suzhou, Jiangsu, China). PTM4 was prepared in the laboratory and stored at 4 °C [49]. *Fusarium* sp. 400226 was earlier purified from *Tadehagi triquetrum* (L.) Ohashi and now preserved in the China Pharmaceutical Culture Collection (http://www.cpcc.ac.cn/fungus/?id=5095, accessed on 1 February 2007).

### 4.2. Microbial Biocatalysis

#### 4.2.1. Cultivation of the Active Strain on a PDA Slant

Approximately 5 μL of CPCC 400226 preserved in frozen glycerol (15%, *v*/*v*) at −80 °C was withdrawn and plated on a PDA slant containing ~5 mL of the medium. The PDA slant was then placed in a thermostatic incubator (MJX-270 type, Ningbo Jiangnan Instrument Factory, Ningbo, Jiangsu, China) and cultured at 28 °C for seven days.

#### 4.2.2. Preparation of the PDB Seed Culture

The PDB seed culture was prepared using previously reported methods [36]. In brief, the strain was washed with sterile water. The resulting fresh liquid was immediately transferred into PDB medium. A PDB seed suspension was prepared by thermostatic cultivation at 30 °C and 200 rpm for 48 h.

#### 4.2.3. Biocatalysis of DZW by *Fusarium* sp. CPCC 400226

About 20 mL of basic SmF medium, including 33.3 g/L of DZW, 2.39 g/L of Na_2_HPO_4_·12H_2_O, and 8.17 g/L of KH_2_PO_4_ (pH 6.0), was transferred into a 125 mL flask. After heat sterilization, biocatalysis of DZW via the SmF of *Fusarium* sp. CPCC 400226 was initiated. Unless otherwise mentioned, the inoculum size, cultivation temperature, fermentation period, and agitation were 0.5 mL, 30 °C, seven days, and 200 rpm, respectively.

### 4.3. Products Analysis

A standard experimental procedure, established in this laboratory, was used for the treatment of the fermentation broth and quantitative analysis of diosgenin [36]. The diosgenin yield was determined by the following equation:

*Y* = C_after_/C_pro_ × 100% (2)

where *Y* is the diosgenin yield (%), C_after_ is the diosgenin content in SmF broth (mg/mL), and C_pro_ is the DZW concentration in SmF medium (mg/mL).

### 4.4. Design of Experiments

#### 4.4.1. Plackett–Burman Design (PBD)

The following first-order polynomial model was employed to fit the PBD:

(3) $$Y=\beta_{0}+\overset{k}{\underset{i=1}{\sum }}\beta_{i}X_{i}$$

where the predicted response is indicated by the value of *Y*, the model intercept, linear coefficient, and level of the independent variable, and the number of the involved variables are presented by the values of *β*_0_, *β_i_*, *X_i_*, and *k*, respectively. *Y* is the yield of diosgenin (%). *X* is the independent variable.

To screen and select the main medium components that have a significant effect on the yield of diosgenin via the microbial biocatalysis of DZW by *Fusarium* sp. CPCC 400226, the following seven factors were evaluated by 12 runs of the PBD (Table 5): seven medium components were tested: Tween-80, vitamin, amino acid, PTM4, yeast extract, wheat bran, and phosphate. In the PBD model, the variables for the component were denoted by factors *A* (Tween-80 (Tween)), *B* (vitamin (Vit)), *C* (amino acid (AA)), *D* (PTM4 (PTM)), *E* (yeast extract (YE)), *F* (wheat bran (WHB)), and *G* (phosphate (PS)). In addition, four unassigned variables were introduced: DV1, DV2, DV3, and DV4. These were used as dummy variables and denoted by factors *J*, *K*, and *L*, respectively. Each factor was tested under level −1 for low levels and level +1 for high levels.

#### 4.4.2. Box–Behnken Design (BBD)

The following second-order polynomial model was employed to fit the BBD:

*Y* = *β*_0_ + *β*_1_*X*_1_ + *β*_2_*X*_2_ + *β*_3_*X*_3_ + *β*_12_*X*_1_*X*_2_ + *β*_13_*X*_1_*X*_3_ + *β*_23_*X*_2_*X*_3_ + *β*_11_*X*_1_^2^ + *β*_22_*X*_2_^2^ + *β*_33_*X*_3_^2^ (4)

where the predicted response is indicated by the value of *Y*, and *β*_0_ is a constant term. *X*_1_, *X*_2_, and *X*_3_ are significant independent variables. The linear regression coefficients are presented by the values of *β*_1_, *β*_2_, and *β*_3_. The quadratic regression coefficients are presented by the values of *β*_11_, *β*_22_, and *β*_33_. The interactive regression coefficients are presented by the values of *β*_12_, *β*_13_, and *β*_23_. *Y* is the yield of diosgenin (%).

To determine the significant effects and the mutual factor interactions between the selected factors, the following three significant factors were tested by 15 runs of the BBD (Table 6): wheat bran, phosphate, and Tween-80. In the BBD model, the variables for the significant medium component were denoted by factors *X*_1_ (WHB), *X*_2_ (PS), and *X*_3_ (Tween), respectively. Each factor was tested under three different levels: central point 0, factorial points −1, and factorial points +1.

### 4.5. Model Verification

The optimum conditions were used for the microbial biocatalysis of DZW by *Fusarium* sp. CPCC 400226 via SmF. The primary SmF conditions of the cultivation temperature and fermentation period were 30 °C and 12 days, respectively.

### 4.6. Data Analysis

Three replicates were performed, and the results were presented as mean ± S.D. Design-Expert software (trial version, Minneapolis, MN, USA) was used for statistical analysis and graph plotting. A *p*-value of <0.05 was considered statistically significant.

## 5. Conclusions

*Fusarium* sp. is one of the fungal strains that can biotransform DZW and produce diosgenin. By taking *Fusarium* sp. CPCC 400226 as a typical example, the combined PBD–BBD method was successfully used for developing a new medium in SmF. With this medium, a final diosgenin yield of 2.23% was obtained, representing a significant increase in diosgenin production. Low-cost components (wheat bran, phosphate, and Tween-80) were used for microbial biocatalysis, which led to a decrease in cost but an increase in production and productivity. We suggest that the statistical design offers an efficient strategy for medium development for diosgenin production. The fundamental information provided in this work gives a basis for further investigation on the large-scale microbial biocatalysis of DZW by *Fusarium* strains in SmF. Moreover, the current study provides critical information for clean and efficient diosgenin production in an acid-free and wastewater-free manner.

## Figures and Tables

**Table 1:** Diosgenin yield under various submerged fermentation (SmF) conditions in Plackett–Burman design (PBD) matrices.

Run Order	*A*: Tween	*B*: Vit	*C*: AA	*D*: PTM	*E*: YE	*F*: WHB	*G*: PS	*H*: DV1	*J*: DV2	*K*: DV3	*L*: DV4	Diosgenin Yield (%)	
												Predicted Value	Experimental Value
1	+1	−1	+1	+1	−1	+1	+1	+1	−1	−1	−1	2.06	1.99
2	−1	+1	−1	+1	+1	−1	+1	+1	+1	−1	−1	0.63	0.49
3	+1	+1	−1	−1	−1	+1	−1	+1	+1	−1	+1	1.41	1.32
4	−1	−1	−1	+1	−1	+1	+1	−1	+1	+1	+1	1.71	1.78
5	−1	−1	+1	−1	+1	+1	−1	+1	+1	+1	−1	1.08	1.03
6	+1	−1	−1	−1	+1	−1	+1	+1	−1	+1	+1	1.02	1.08
7	−1	−1	−1	−1	−1	−1	−1	−1	−1	−1	−1	0.10	0.11
8	−1	+1	+1	−1	+1	+1	+1	−1	−1	−1	+1	1.76	1.82
9	+1	+1	−1	+1	+1	+1	−1	−1	−1	+1	−1	1.25	1.34
10	+1	+1	+1	−1	−1	−1	+1	−1	+1	+1	−1	1.13	1.16
11	+1	−1	+1	+1	+1	−1	−1	−1	+1	−1	+1	0.30	0.29
12	−1	+1	+1	+1	−1	−1	−1	+1	−1	+1	+1	0.06	0.12

**Table 2:** Statistical analysis of the PBD model.

Source	SS	Df	MS	*F*-Value	*p*-Value	C%
Model	4.75	7	0.6788	47.1	0.0011	-
*A* (Tween)	0.2783	1	0.2783	19.3	0.0117	5.79
*B* (Vit)	0.0001	1	0.0001	<1.0	0.9540	0.0011
*C* (AA)	0.0066	1	0.0066	<1.0	0.5369	0.14
*D* (PTM)	0.0210	1	0.0210	1.45	0.2942	0.44
*E* (YE)	0.0145	1	0.0145	1.00	0.3732	0.30
*F* (WHB)	3.0300	1	3.0300	210.1	0.0001	62.93
*G* (PS)	1.4000	1	1.4000	97.5	0.0006	29.21
Residual	0.0576	4	0.0144			
Cor Total	4.81	11				

Model summary: R^2^, 0.9880; adjusted R^2^, 0.9670; predicted R^2^, 0.8921; C%, contribution %. *p*-value < 0.05, 5% significant level. SS, sum of squares; Df, degree of freedom; MS, mean sum of squares.

**Table 3:** Diosgenin yield under various SmF conditions in Box–Behnken design (BBD) matrices.

Run Order	*X*_1_: WHB	*X*_2_: PS	*X*_3_: Tween	Diosgenin Yield (%)	
				Predicted Value	Experimental Value
1	0	−1	−1	1.40	1.42
2	0	+1	+1	1.99	1.97
3	0	−1	+1	1.51	1.51
4	+1	0	+1	1.48	1.50
5	−1	+1	0	1.22	1.23
6	+1	−1	0	1.72	1.71
7	−1	−1	0	0.39	0.39
8	0	0	0	1.96	1.96
9	+1	+1	0	1.95	1.95
10	−1	0	+1	0.72	0.73
11	0	0	0	1.96	1.93
12	0	+1	−1	1.97	1.97
13	0	0	0	1.96	1.98
14	+1	0	−1	1.69	1.68
15	−1	0	−1	0.39	0.37

**Table 4:** Statistical analysis of the BBD model.

Source	SS	Df	MS	*F*-Value	*p*-Value
Model	4.52	9	0.5026	812.8	<0.0001
*X*_1_-WHB	2.12	1	2.12	3431.2	<0.0001
*X*_2_-PS	0.5460	1	0.5460	883.0	<0.0001
*X*_3_-Tween	0.0091	1	0.0091	14.5	0.0121
*X*_1_ *X*_2_	0.0900	1	0.0900	145.6	<0.0001
*X*_1_ *X*_3_	0.0729	1	0.0729	117.9	0.0001
*X*_2_ *X*_3_	0.0020	1	0.0020	3.3	0.1301
*X*_1_^2^	1.5200	1	1.5200	2461.8	<0.0001
*X*_2_^2^	0.0001	1	0.0001	<1.0	0.6929
*X*_3_^2^	0.2209	1	0.2209	357.2	<0.0001
Residual	0.0031	5	0.0006		
Lack of Fit	0.0018	3	0.0006	1.0	0.5465
Pure Error	0.0013	2	0.0006		
Cor Total	4.53	14			

Model summary: R^2^, 0.9993; adjusted R^2^, 0.9981; predicted R^2^, 0.9929.

**Table 5:** Factors and their levels examined in the PBD.

Variables	Symbol	Actual Experimentation Value	
		Low (−1)	High (+1)
Tween-80	*A* (Tween)	1.5	2.5
Vitamin	*B* (Vit)	0.8	1.2
Amino acid	*C* (AA)	0.8	1.2
PTM4	*D* (PTM)	1.0	1.5
Yeast extract	*E* (YE)	8	12
Wheat bran	*F* (WHB)	16	24
Phosphate	*G* (PS)	6.4	9.6

**Table 6:** Factors and their levels examined in the BBD.

Variables	Symbol	Levels		
		−1	0	+1
Wheat bran	*X*_1_ (WHB)	16	20	24
Phosphate	*X*_2_ (PS)	6.4	10.0	9.6
Tween-80	*X*_3_ (Tween)	1.5	2.0	2.5

## Data Availability

The data presented in this study are available in the main body text and the Supplementary Material of the current article.

## Supplementary Materials

The following are available online at https://www.mdpi.com/article/10.3390/ph14050390/s1: Figure S1. Diagnostic plots of the BBD model adequacy for diosgenin yield; Figure S2. The overlay plot of perturbation for diosgenin yield.
