# Supplementary material for: Screening and Selection of a New Medium for Diosgenin Production via Microbial Biocatalysis of Fusarium sp
Source: Pharmaceuticals (Basel). 2021 Apr 21;14(5):390. doi: 10.3390/ph14050390 (PMC8143133; doi:10.3390/ph14050390)

Supplementary Materials

Screening and selection of a new medium for diosgenin production via microbial biocatalysis of *Fusarium* sp.

Wancang Liu [1], Haibo Xiang [2], Tao Zhang [1], Xu Pang [1], Jing Su [1], Hongyu Liu [1], Baiping Ma [3] and Liyan Yu [1,*]

[1] Division for Medicinal Microorganisms Related Strains CAMS Collection Center of Pathogenic Microorganisms, Institute of Medicinal Biotechnology, Chinese Academy of Medical Sciences & Peking Union Medical College, 100050 Beijing, China; liuwancang@imb.pumc.edu.cn (W.L.); zhangt@cpcc.ac.cn (T.Z.) pangxu@imb.pumc.edu.cn (X.P.); sujing@cpcc.ac.cn (J.S.); hyliu@cpcc.ac.cn (H.L.)

[2] State Key Laboratory of Biocatalysis and Enzyme Engineering, School of Life Sciences, Hubei University, 430011 Wuhan, China; xhb2086@hubu.edu.cn

[3] Beijing Institute of Radiation Medicine, 100850 Beijing, China; mabaiping@sina.com

* Correspondence: yly@cpcc.ac.cn; Tel.: +86 10 63187118

Figure S1: Diagnostic plots of the BBD model adequacy for diosgenin yield. (a) The plot of predicted values versus experimental values. (b) The plot of normal % probability. (c) The plot of internally studentized residuals.

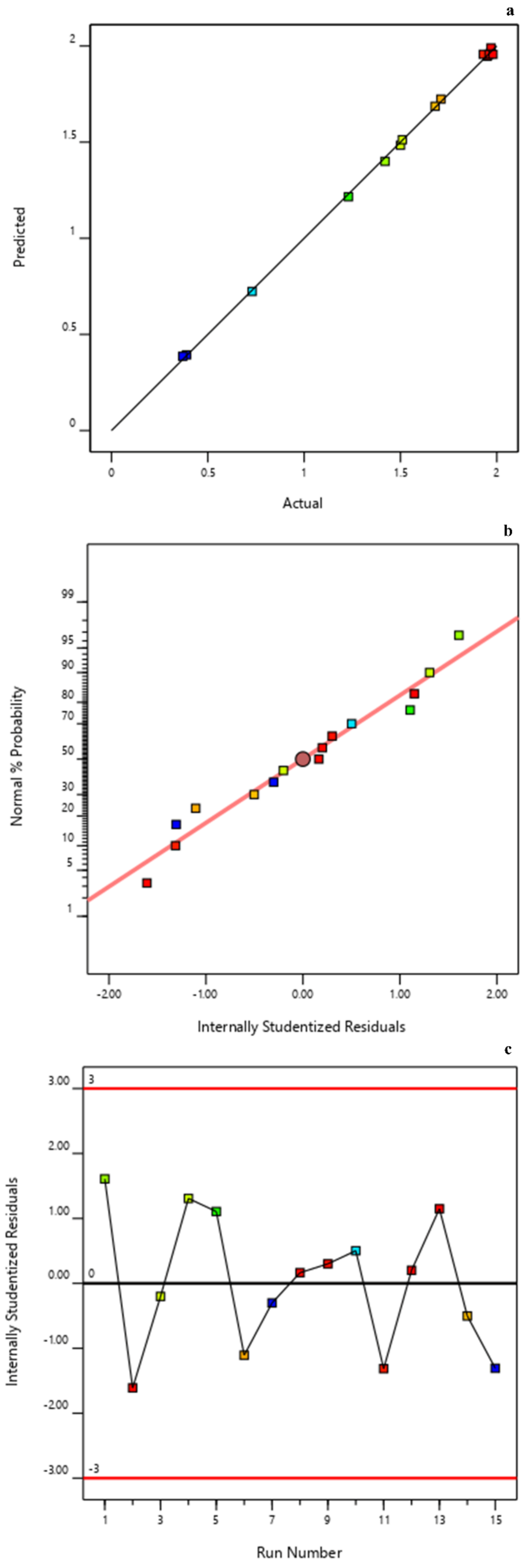

Figure S2: The overlay plot of perturbation for diosgenin yield. A, wheat bran. B, phosphate. C, Tween-80.

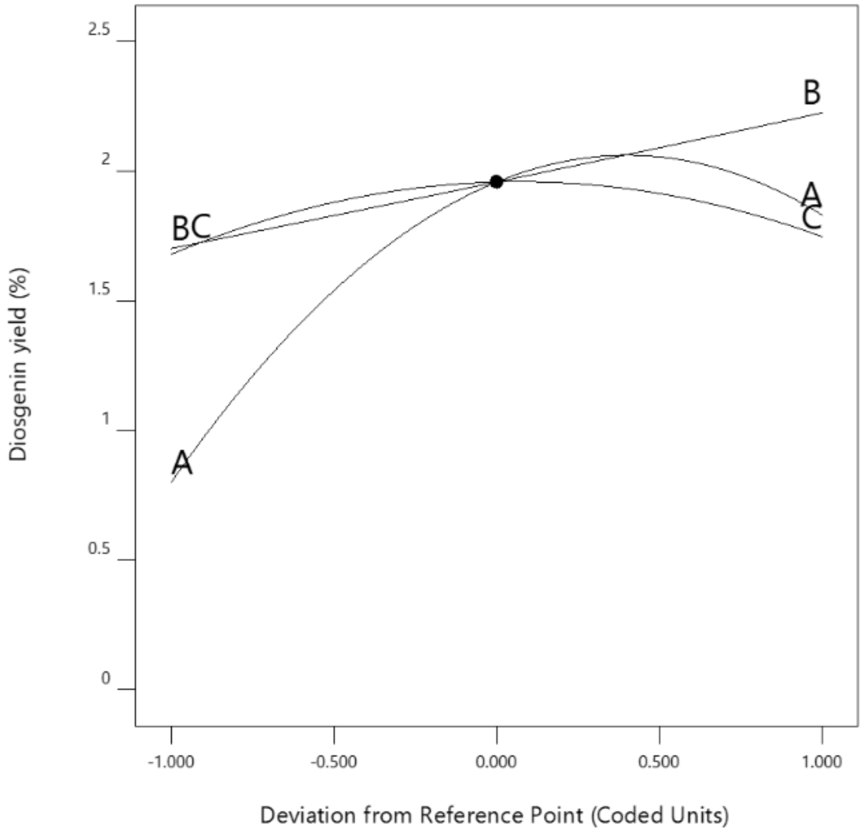

Supplement: Supplementary file 1 [file pharmaceuticals-14-00390-s001.zip › pharmaceuticals-1176303-supplementary.pdf]
